# Rising cesarean deliveries among apparently low-risk mothers at university teaching hospitals in Jordan: analysis of population survey data, 2002–2012

**DOI:** 10.9745/GHSP-D-14-00027

**Published:** 2014-05-13

**Authors:** Rami Al Rifai

**Affiliations:** a Graduate School of Tokyo Medical and Dental University, Division of Public Health, Department of International Health and Medicine, Tokyo, Japan

## Abstract

**Background:**

Cesarean delivery conducted without medical indication places mothers and infants at risk for adverse outcomes. This study assessed changes in trends of, and factors associated with, cesarean deliveries in Jordan, from 2002 to 2012.

**Methods:**

Data for ever-married women ages 15–49 years from the 2002, 2007, and 2012 Jordan Population and Family Health Surveys were used. Analyses were restricted to mothers who responded to a question regarding the hospital-based mode of delivery for their last birth occurring within the 5 years preceding each survey (2002, N = 3,450; 2007, N = 6,307; 2012, N = 6,365). Normal birth weight infants and singleton births were used as markers for births that were potentially low risk for cesarean delivery, because low/high birth weight and multiple births are among the main obstetric variables that have been documented to increase risk of cesareans. Weighted descriptive and multivariate analyses were conducted using 4 logistic regression models: (1) among all mothers; and among mothers stratified (2) by place of delivery; (3) by birth weight of infants; and (4) by singleton vs. multiple births.

**Results:**

The cesarean delivery rate increased significantly over time, from 18.2% in 2002, to 20.1% in 2007, to 30.3% in 2012. Place of delivery, birth weight, and birth multiplicity were significantly associated with cesarean delivery after adjusting for confounding factors. Between 2002 and 2012, the rate increased by 99% in public hospitals vs. 70% in private hospitals; by 93% among normal birth weight infants vs. 73% among low/high birth weight infants; and by 92% among singleton births vs. 29% among multiple births. The changes were significant across all categories except among multiple births. Further stratification revealed that the cesarean delivery rate was 2.29 times higher in university teaching hospitals (UTHs) than in private hospitals (*P*< .001), and 2.31 times higher than in government hospitals (*P*< .001). Moreover, in UTHs, the rate was higher among normal birth weight infants (adjusted OR = 2.15) and singleton births (adjusted OR = 2.39).

**Conclusion:**

The rising cesarean delivery rate among births that may have been at low risk for cesarean delivery, particularly in UTHs, indicates that many cesarean deliveries may increasingly be performed without any medical indication. More vigilant monitoring of data from routine health information systems is needed to reduce unnecessary cesarean deliveries in apparently low-risk groups.

## Abstract (precis)

Cesarean deliveries nationally in Jordan have increased to 30%, including substantial increases among births that are likely low risk for cesarean delivery for the most part. This level is double the threshold that WHO considers reasonable.

## INTRODUCTION

Worldwide, cesarean delivery is one of the most common surgeries performed in modern obstetrics.^1^ The surgery is intended to save the lives of mothers and newborns, as in cases of dystocia, breech presentation, multiple births, anticipated low/high birth weight, and fetal distress.^2^^,^^3^ But cesarean deliveries conducted without any medical indication place mothers and infants at risk for unfavorable outcomes. For example, compared with vaginal delivery, cesarean delivery is associated with increased risk of blood transfusion, hysterectomy, maternal and child death,^4^^–^^6^ uterine rupture, placenta accreta, and placenta previa in a subsequent pregnancy.^7^^,^^8^ In addition, cesarean deliveries cost more and require longer hospitalization than vaginal deliveries.^9^

The World Health Organization (WHO) considers a population-based rate of cesarean deliveries over 15% unreasonable.^10^ A recent study found that half of 137 countries have exceeded this recommended threshold.^11^ A global survey conducted by WHO in 24 countries over 25 years showed that about 26% of all health facility-based deliveries were cesarean, and in 23 countries, rates of cesarean delivery without medical indication ranged between 0.01% and 2%.^5^ In Jordan, the last study examining trends in cesarean delivery rates using population-based data revealed a consistent increase in the rate.^12^

WHO considers 15% the threshold for a reasonable population-based cesarean delivery rate.

Jordan has one of the most modern health care infrastructures in the Middle East. The health system consists of 3 major sectors—public, private, and donors—that cover primary, secondary, and tertiary health care services. The public sector consists of health facilities governed by the Ministry of Health (MOH), Royal Medical Services (RMS), and University Teaching Hospitals (UTHs).^13^ Only tertiary health care facilities (hospitals) have the capacity to perform major surgical procedures such as cesarean deliveries.

According to the Jordan Population and Family Health Survey (JPFHS), almost all women (99%) in the country receive antenatal care (ANC) from a skilled provider, most commonly from a doctor (96%). Nearly all women (91%) have an ANC visit before their fourth month of pregnancy, and 78% of women make 7 or more ANC visits throughout their pregnancy.^14^ Virtually all births (99%) occur in a health facility; 3 births of every 4 are delivered by a doctor, and 1 birth of every 4, by a nurse or midwife.^14^ The maternal mortality ratio increased from 53 maternal deaths per 100,000 live births in 2008 to 63/100,000 in 2010, while the neonatal mortality rate persisted at rate of 12 deaths/1,000 live births between 2009 and 2013.^15^^,^^16^

The objectives of this study were to examine trends in, and factors associated with, cesarean deliveries in Jordan, from 2002 to 2012, using data from nationally representative surveys of ever-married women ages 15–49 years.

## METHODS

### Data Source and Sampling

This study used nationally representative data from the 2002, 2007, and 2012 JPFHS, which is part of the worldwide Demographic and Health Surveys (DHS) program.^14^

In total, 28,234 ever-married women ages 15–49 years participated in the 3 JPFHS survey rounds (Figure). Women who did not report giving birth over the prior 5 years or who reported that they delivered their last birth at home or abroad were excluded, resulting in a final sample of 16,774 women, or a weighted sample of 16,122 women (3,450 from the 2002 survey; 6,307 from 2007; and 6,365 from 2012).

**FIGURE.**
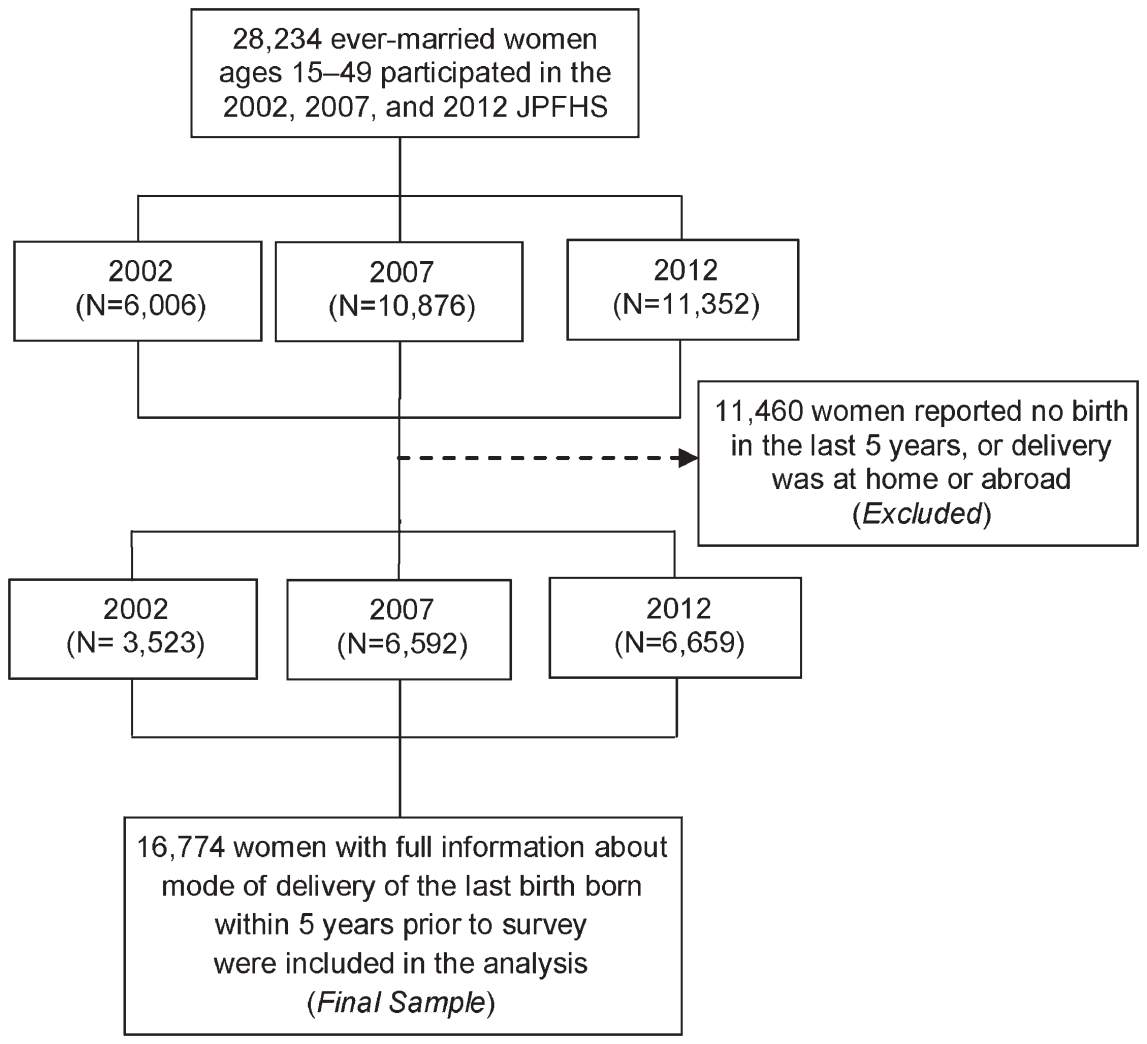
Selection (Unweighted Numbers) Abbreviation: JPFHS, Jordan Population and Family Health Survey.

### Measurements

#### Outcome Variable

The outcome of interest was hospital-based mode of delivery for the last birth; cesarean delivery was reported as a binary response (yes/no).

#### Sociodemographic Variables

The analysis included information about different sociodemographic characteristics that may be associated with cesarean delivery:

- Woman's age reported in 5 groups (15–19, 20–24, 25–29, and ≥ 30 years)
- Place of residence (urban/rural)
- Geographic region (central, north, or south)
- Mother's and partner's education level (no education, primary, secondary, or higher education)
- Employment status (mothers with any income-producing profession were reported as employed)
- Place of delivery (public hospitals governed by the MOH, RMS, or UTHs, or private hospitals)
- Wealth index constructed using data on household assets and categorized into quintiles (poorest, poorer, middle, richer, and richest)^14^^,^^17^

#### Obstetric Variables

- Mother's age at first birth was reported in 4 groups: 12–18, 19–24, 25–30, or > 30 years.
- Parity was reported in 3 groups: 1, 2, or 3 or more children.
- Since low or high birth weight and multiple births are among the main obstetric variables documented in the literature to increase risk of cesarean delivery,^12^^,^^18^^,^^19^ these 2 variables were used in this analysis as markers for births that were potentially at low risk for cesarean delivery (while recognizing that this may not always be the case). Women who delivered either singleton or normal birth weight infants (2,500 to 3,999 grams) were categorized as low risk for cesarean delivery while women who delivered either multiple or low/high birth weight infants (< 2,500 grams or ≥ 4,000 grams) were categorized as high risk.^12^

### Statistical Analysis

Frequencies and percentages for each measured characteristic by year of survey were recorded. Rates of cesarean delivery for the last birth born within the 5 years prior to the survey were compared between the 2002, 2007, and 2012 surveys, and the potential difference in the cesarean rate for each measured variable was evaluated for each survey, separately, by using chi-square tests.

To document trends in the cesarean rate over a period of 10 years, the first survey conducted in 2002 was set as a reference in 4 binary logistic regression models, which estimated odds ratios (ORs) and adjusted odds ratios (aORs) as well as 95% confidence intervals (CIs) for significance testing. The first model was run among all mothers together and the other 3 models were run for mothers stratified by: (1) place of delivery (public or private), (2) birth weights, and (3) birth multiplicity (singleton vs. multiple births).

To assess whether place of delivery was associated with changes in trends of cesarean deliveries, all subjects were further stratified according to birth weights and birth multiplicity in public and private hospitals, separately, as well as by type of public hospital (government hospitals vs. UTHs) for those who reported delivering in a public hospital. In order to control for potential confounding effects, even when there was no association with cesarean delivery, all covariates under analysis (sociodemographic and obstetric) were entered simultaneously in the multivariate logistic regression models.

Logistic regression models were also used to assess the association between measured covariates with cesarean delivery and to investigate the independent association of the year of the survey on cesarean delivery after merging the 3 datasets. *P* value for the trend was assessed for each model by entering the year of the survey as a continuous variable.^20^ All analyses were conducted using SPSS version 18.0. The level of statistical significance for all analysis was set at α = .05.

## RESULTS

### Descriptive Profile of the Mothers

Overall, 57.4% of mothers in the 3 datasets combined were ≥ 30 years old. The majority lived in urban areas and had secondary or higher education. In 2002, 10.1% of the mothers were employed compared with 15.5% in 2012 (Table 1).

**TABLE 1.** Background Characteristics of Survey Respondents by Year of Survey, Weighted Numbers (Percentages)

**Characteristics**	**2002 (N = 3,450)**	**2007 (N = 6,307)**	**2012 (N = 6,365)**
**SOCIODEMOGRAPHIC**			
Age, y			
15–19	55 (1.6)	123 (1.9)	135 (2.1)
20–24	515 (14.9)	974 (15.4)	854 (13.4)
25–29	884 (25.6)	1654 (26.2)	1669 (26.2)
≥ 30	1997 (57.9)	3556 (56.4)	3707 (58.2)
Place of Residence			
Urban	2705 (78.4)	5298 (84.0)	5198 (81.7)
Rural	746 (21.6)	1009 (16.0)	1167 (18.3)
Geographic Region			
Central	2191 (63.5)	3908 (62.0)	3935 (61.8)
North	930 (27.0)	1844 (29.2)	1821 (28.6)
South	329 (9.5)	555 (8.8)	609 (9.6)
Mother's Education			
No education	128 (3.7)	147 (2.3)	114 (1.8)
Primary	277 (8.0)	337 (5.3)	373 (5.9)
Secondary	2087 (60.5)	3885 (61.6)	3825 (60.1)
Higher	959 (27.8)	1939 (30.7)	2053 (32.2)
Husband's Education			
No education	65 (1.9)	91 (1.5)	59 (0.9)
Primary	371 (10.8)	665 (10.6)	661 (10.4)
Secondary	1980 (57.4)	3793 (60.3)	4000 (62.8)
Higher	1034 (30.0)	1745 (27.7)	1645 (25.8)
Missing (Do not know)		12 (0.2)	1 (0.02)
Employment			
Unemployed	3101 (89.9)	5526 (87.6)	5381 (84.5)
Employed	350 (10.1)	781 (12.4)	985 (15.5)
Place of Delivery			
Public sector	2229 (64.6)	4036 (64.0)	4230 (66.5)
Private sector	1221 (35.4)	2271 (36.0)	2135 (33.5)
Wealth Index			
Poorest	786 (22.8)	1462 (23.2)	1313 (20.6)
Poorer	881 (25.5)	1475 (23.4)	1365 (21.4)
Middle	740 (21.4)	1368 (21.7)	1437 (22.6)
Richer	594 (17.2)	1131 (17.9)	1291 (20.3)
Richest	450 (13.0)	872 (13.8)	960 (15.1)
**OBSTETRIC**			
Age at First Birth, y			
12–18	687 (19.9)	1110 (17.6)	917 (14.4)
19–24	2010 (58.3)	3661 (58.1)	3747 (58.9)
25–30	645 (18.7)	1289 (20.4)	1369 (21.5)
> 30	107 (3.1)	246 (3.9)	332 (5.2)
Parity in Last 5 Years			
1	1881 (54.5)	3531 (56.0)	3733 (58.6)
2	1263 (36.6)	2254 (35.7)	2182 (34.3)
≥ 3	307 (8.9)	522 (8.3)	451 (7.1)
Birth Weight, g			
Normal (2500–3999)	2618 (75.9)	4916 (77.9)	4907 (77.1)
Low or High (< 2500 or ≥ 4000)	832 (24.1)	1391 (22.1)	1458 (22.9)
Birth Multiplicity			
Singleton birth	3371 (97.7)	6185 (98.1)	6225 (97.8)
Multiple birth	80 (2.3)	123 (1.9)	140 (2.2)

All data are reported as No. (%).

Approximately two-thirds (65.1%) of deliveries were in public hospitals with no significant changes in that percentage over the study period. The percentage of women belonging to poor (“poorest” and “poorer”) households declined by 8.5 percentage points between 2002 and 2012, with a corresponding 5.2 percentage point increase in those belonging to rich households (“richer” and “richest”).

First birth occurred at age 19–24 years among 58.5% of mothers, and most had 1 or 2 children. From 2002 to 2012, there was no significant change in the percentage of mothers reporting low/high birth weight infants or multiple births.

### Trends in the Cesarean Delivery Rate

The cesarean delivery rate increased over time, from 18.2% in 2002 to 30.3% in 2012 (Table 2). Between 2007 and 2012, the rate increased by 10.2 percentage points compared with an increase of only 1.9 percentage points from 2002 to 2007.

**TABLE 2.** Cesarean Delivery Rate by Year of Survey and Background Characteristics, Weighted Percentages

**Characteristics**	**2002**		**2007**		**2012**		****	****
	**%**	***P* Value**	**%**	***P* Value**	**%**	***P* Value**	**Percentage Point Difference^a^**	***P* Value for Trend^b^**
***Overall Rate***	***18.2***		***20.1***		***30.3***			
Age, y		.001		<.001		<.001		.53
15–19	14.5		20.3		22.2		7.7	
20–24	14.6		11.1		25.2		10.6	
25–29	15.5		16.9		28.0		12.5	
≥ 30	20.5		24.0		32.8		12.3	
Place of Residence		.33		.45		.12		.74
Urban	18.6		20.3		30.7		12.1	
Rural	17.0		19.2		28.4		11.4	
Geographic Region		.008		.18		.06		.37
Central	19.7		20.2		31.2		11.5	
North	15.1		19.1		28.2		13.1	
South	17.3		22.7		30.9		13.6	
Mother's Education		.01		.001		<.001		.75
No education	22.0		24.5		23.5		1.5	
Primary	23.1		24.9		40.2		17.1	
Secondary	16.6		18.6		29.0		12.4	
Higher	19.9		22.0		31.3		11.4	
Partner's Education		.13		.44		<.001		.48
No education	23.1		20.7		28.8		5.7	
Primary	16.9		21.5		33.0		16.1	
Secondary	17.3		19.4		28.1		10.8	
Higher	20.3		20.9		34.6		14.3	
Employment		.02		<.001		<.001		.02
Unemployed	17.7		19.4		28.5		10.8	
Employed	22.6		24.8		40.1		17.5	
Place of Delivery		.004		.005		.02		.53
Public sector	16.8		19.0		29.4		12.6	
Private sector	20.8		22.0		32.2		11.4	
Wealth Index		.18		<.001		<.001		.47
Poorest	17.4		17.0		28.8		11.4	
Poorer	16.3		17.1		25.4		9.1	
Middle	18.1		19.2		33.3		15.2	
Richer	20.0		24.0		31.0		11.0	
Richest	21.1		26.5		34.0		12.9	
Parity in Last 5 Years		<.001		.02		.01		.01
1	20.1		21.3		30.0		9.9	
2	14.8		18.5		29.5		14.7	
≥ 3	20.6		18.4		36.6		16.0	
Age at First Birth, y		<.001		<.001		<.001		<.001
12–18	16.9		16.8		24.3		7.4	
19–24	16.7		17.0		27.0		10.3	
25–30	20.3		28.2		34.5		14.2	
> 30	43.9		38.5		66.9		23.0	
Birth Weight, g		<.001		<.001		<.001		.85
Normal (2500–3999)	16.3		18.1		28.4		12.1	
Low/High (< 2500 or ≥ 4000)	24.2		27.1		36.6		12.4	
Birth Multiplicity		<.001		<.001		<.001		<.001
Singleton birth	17.3		19.3		29.5		12.2	
Multiple birth	60.0		59.3		65.7		5.7	

a Percentage point difference between 2002 and 2012.

b *P* value for the trend was obtained from logistic regression models by using outcome and explanatory variables as continuous variables and including interaction terms between survey year and each tested explanatory variable for the years 2002 and 2012.

The cesarean delivery rate in Jordan increased from 18% in 2002 to 30% in 2012.

Age, mother's education, and place of delivery persisted as significant factors in the cesarean delivery rate during each survey round (*P* < .05 within each indicator for each year) (Table 2). The rate generally increased over time for all categories within these indicators, thus displaying similar trends (*P* > .05 for the trend over years for these indicators). The largest increases over time were observed among mothers aged 25–29 years and ≥ 30 years and among mothers with primary education.

Mother's employment, parity, and age at first birth were also all significant factors in the cesarean delivery rate during each survey round, and the increases over time were also significant. In each category, the largest increases were among employed mothers (17.5 percentage point increase between 2002 and 2012), mothers with 3 or more children (23 percentage point increase), and mothers whose age at first birth was over 30 years (23 percentage point increase).

Wealth was a significant factor in the cesarean delivery rate in the 2007 and 2012 survey years. Mothers in the poor wealth categories (“poorest” and “poorer”) generally had lower cesarean delivery rates than those in the rich categories (“richer” and “richest”), but the largest increase in cesarean deliveries over time was among mothers belonging to middle-income households (15.2 percentage point increase).

During the study period, the increase in the cesarean delivery rate among the normal birth weight group was the same as that of the low/high birth weight group (about a 12 percentage point increase), but the rate increased more among singleton than among multiple births (12.2 percentage points vs. 5.7 percentage points, respectively; *P*< .001) (Table 2).

### Trends in the Cesarean Delivery Rate Stratified by Place of Delivery, Birth Weight, and Birth Multiplicity

Place of delivery, birth weight, and birth multiplicity were significantly associated with cesarean delivery in the crude analysis and retained their statistical significance after adjustment for all covariates (Table 3). Multivariate models from the 3 combined datasets showed that the cesarean delivery rate increased significantly over time, by 13% between 2002 and 2007, and by 90% between 2002 and 2012 (*P*< .001).

**TABLE 3.** Bivariate and Multivariate Logistic Regression Analysis on Trends in the Cesarean Delivery Rate, Among All Mothers and Stratified by Place of Delivery, Birth Weight, and Birth Multiplicity According to Year of Survey

	**No. (%)**	**OR (95% CI)**	**aOR (95% CI)**
**Among All Mothers**			
2002	629 (18.2)	1.00	1.00
2007	1267 (20.1)	1.13 (1.01–1.25)*	1.13 (1.01–1.26)*
2012	1929 (30.3)	1.95 (1.76–2.16)^***^	1.90 (1.71–2.11)^***^
**By Place of Delivery**			
Public Sector			
2002	375 (16.8)	1.00	1.00
2007	768 (19.0)	1.16 (1.01–1.33)*	1.15 (1.00–1.33)*
2012	1242 (29.4)	2.05 (1.80–2.34)^***^	1.99 (1.74–2.28)^***^
Private Sector			
2002	254 (20.8)	1.00	1.00
2007	499 (22.0)	1.07 (0.90–1.27)	1.06 (0.88–1.26)
2012	687 (32.2)	1.80 (1.53–2.12)^***^	1.70 (1.43–2.02)^***^
**By Birth Weight**			
Normal			
2002	428 (16.3)	1.00	1.00
2007	890 (18.1)	1.13 (0.99–1.28)	1.11 (0.98–1.26)
2012	1395 (28.4)	2.03 (1.80–2.29)^***^	1.93 (1.71–2.19)^***^
Low/High			
2002	201 (24.2)	1.00	1.00
2007	377 (27.1)	1.17 (0.96–1.42)	1.13 (0.93–1.39)
2012	534 (36.6)	1.81 (1.50–2.20)^***^	1.73 (1.42–2.10)^***^
**By Birth Multiplicity**			
Singleton Birth			
2002	582 (17.3)	1.00	1.00
2007	1194 (19.3)	1.15 (1.03–1.28)*	1.14 (1.02–1.27)*
2012	1838 (29.5)	2.01 (1.81–2.23)^***^	1.92 (1.72–2.13)^***^
Multiple Birth			
2002	48 (60.0)	1.00	1.00
2007	73 (589.3)	0.99 (0.55–1.75)	0.98 (0.49–1.95)
2012	92 (65.7)	1.28 (0.72–2.26)	1.29 (0.64–2.58)

Abbreviations: aOR, adjusted odds ratio (for all covariates under analysis); CI, confidence interval; OR, odds ratio.

*P* value for the trend was obtained by entering the survey year as a continuous variable.

^*^P< .05; ^***^ *P*< .001.

Between 2002 and 2012, stratification showed that the cesarean delivery rate increased by 99% in public hospitals vs. 70% in private hospitals; the rate increased by 93% among the normal birth weight group vs. 73% among the low/high birth weight group. During the same period, the cesarean delivery rate increased significantly by 92% among singleton births (*P*< .001), whereas it increased by only 29% among multiple births (*P* = .43).

Over the past decade, the cesarean delivery rate in Jordan increased by 93% among normal birth weight infants vs. 73% among low/high birth weight infants.

Table 4 shows trends in cesarean deliveries among all subjects stratified by birth weight and birth multiplicity according to place of delivery. The cesarean delivery rate for mothers of normal birth weight babies in public hospitals was 2.06 times higher in 2012 than in 2002, and in private hospitals, 1.71 times higher. Similarly, the cesarean delivery rate for singleton births in public hospitals was 2.0 times higher in 2012 than in 2002, and in private hospitals, 1.76 times higher. Mothers who delivered multiple births in public hospitals were 3 times more likely to undergo cesarean delivery in 2012 than in 2002. In contrast, in private hospitals, the rate declined by 92% over time (aOR = 0.08; *P* = .03).

**TABLE 4.** Multivariate Logistic Regression Analysis on Trends in the Cesarean Delivery Rate by Place of Delivery, Stratified by Birth Weight and Birth Multiplicity According to Year of Survey

	**Public Hospitals (N = 10,496)**		**Private Hospitals (N = 5,627)**	
	**No. (%)**	**aOR (95% CI)**	**No. (%)**	**aOR (95% CI)**
**By Birth Weight**				
Normal				
2002	247 (14.8)	1.00	181 (19.1)	1.00
2007	525 (17.0)	1.17 (0.99–1.39)	365 (19.9)	1.00 (0.82–1.23)
2012	877 (27.4)	2.06 (1.76–2.42)^***^	519 (30.4)	1.71 (1.41–2.09)^***^
Low/High				
2002	128 (22.9)	1.00	73 (26.7)	1.00
2007	242 (25.3)	1.12 (0.87–1.45)	135 (31.0)	1.22 (0.85–1.75)
2012	366 (35.6)	1.87 (1.46–2.38)^***^	168 (39.1)	1.66 (1.16–2.38)^**^
**By Birth Multiplicity**				
Singleton Birth				
2002	347 (16.0)	1.00	234 (19.6)	1.00
2007	718 (18.2)	1.15 (0.99–1.33)	476 (21.3)	1.08 (0.90–1.29)
2012	1171 (28.3)	2.00 (1.74–2.29)^***^	667 (31.8)	1.76 (1.47–2.09)^***^
Multiple Birth				
2002	28 (50.9)	1.00	20 (80.0)	1.00
2007	49 (60.5)	1.85 (0.78–4.35)	23 (56.1)	0.32 (0.05–2.09)
2012	72 (72.0)	3.05 (1.27–7.33)*	20 (50.0)	0.08 (0.01–0.78)*

Abbreviations: aOR, adjusted odds ratio (for all covariates under analysis); CI, confidence interval.

*P* value for the trend was obtained by entering the survey year as a continuous variable.

^*^P< .05; ^**^ *P<* .01; ^***^ *P*< .001.

Between 2002 and 2012, the cesarean delivery rate rose sharply in UTHs by 22.4 percentage points compared with increases of 11.4 and 11.9 percentage points in private and government hospitals, respectively (Table 5). Overall, the cesarean delivery rate in UTHs was 2.29 times higher than in private hospitals (*P*< .001) and 2.31 times higher than in government hospitals (*P*< .001). Moreover, the odds of performing cesarean deliveries for low-risk groups—mothers of normal birth weight babies (aOR = 2.15, 95% CI = 1.65–2.80) and singletons (aOR = 2.39, 95% CI = 1.88–3.02)—was higher in UTHs than in governmental hospitals, whereas there was no significant difference in the cesarean delivery rate for multiple births between these hospital types.

**TABLE 5.** Multivariate Logistic Regression Analysis on Cesarean Delivery Rate Among Mothers Delivering in Public Hospitals by Year of Survey, Stratified by Birth Weight and Birth Multiplicity According to Type of Public Hospital

	**2002**		**2007**		**2012**		**All Years Combined**	
	**%**	**aOR (95% CI)**	**%**	**aOR (95% CI)**	**%**	**aOR (95% CI)**	**%**	**aOR (95% CI)**
**All Mothers**								
Private	20.8	1.00	22.0	1.00	32.2	1.00	25.6	1.00
Government (public)^a^	16.6	0.86 (0.69–1.07)	18.1	0.87 (0.75–1.02)	28.5	0.91 (0.79–1.04)	21.9	0.90 (0.83–0.99)*
UTH (public)	26.7	1.08 (0.57–2.05)	50.0	4.24 (2.85–6.29)^***^	49.1	1.69 (1.21–2.33)^**^	45.6	2.29 (1.82–2.88)^***^
**Mothers Delivering in Public Hospitals (N = 10,494)**								
Government	16.6	1.00	18.1	1.00	28.5	1.00	21.9	1.00
UTH	26.7	1.19 (0.63–2.27)	50.0	4.20 (2.80–6.28)^***^	49.1	1.69 (1.21–2.37)^**^	45.6	2.31 (1.83–2.92)^***^
**Mothers Delivering in Public Hospitals By Birth Weight**								
Normal Birth Weight								
Government	14.5	1.00	16.2	1.00	26.5	1.00	19.9	1.00
UTH	23.9	1.18 (0.55–2.55)	44.1	3.44 (2.22–5.33)^***^	47.1	1.55 (1.06–2.27)*	42.2	2.15 (1.65–2.80)^***^
Low/High Birth Weight								
Government	22.5	1.00	24.2	1.00	34.7	1.00	28.1	1.00
UTH	35.7	1.24 (0.35–4.41)	83.3	27.50 (6.54–115.5)^***^	57.1	2.30 (1.08–4.88)*	60.6	3.13 (1.82–5.37)^***^
**Mothers Delivering in Public Hospitals By Birth Multiplicity**								
Singleton Birth								
Government	15.7	1.00	17.2	1.00	27.6	1.00	21.0	1.00
UTH	29.6	1.73 (0.91–3.29)	48.7	4.06 (2.70–6.08)^***^	46.7	1.62 (1.15–2.28)^**^	44.7	2.39 (1.88–3.02)^***^
Multiple Birth								
Government	57.1	1.00	59.5	1.00	69.2	1.00	63.0	1.00
UTH	0.0	NA	100.0	NA	100.0	NA	64.7	1.02 (0.31–3.38)

Abbreviations: aOR, adjusted odds ratio (for all covariates under analysis); CI, confidence interval; NA, not applicable; UTH, university teaching hospital.

a Government hospitals include hospitals run by the Ministry of Health or the Royal Medical Services.

^*^P< .05; ^**^ *P<* .01; ^***^ *P*< .001.

The cesarean delivery rate in university teaching hospitals was more than 2 times higher than in private or government hospitals.

Table 6 shows logistic regression analysis on the cesarean delivery rate after combining the 3 datasets. Employed mothers were significantly more likely than unemployed mothers to undergo cesarean delivery (aOR = 1.34, 95% CI = 1.19–1.51; *P*< .001). The odds of undergoing cesarean delivery increased linearly and significantly with increasing wealth and mother's age at first birth. Mothers with only 1 child had higher likelihood of cesarean delivery compared with those with 2 or ≥ 3 children. Low/high birth weight infants also were more likely to have undergone cesareans. However, the strongest predictor that was associated with cesarean delivery was multiple births (aOR = 5.60, 95% CI = 4.20–7.09; *P*< .001).

**TABLE 6.** Logistic Regression Analysis on Cesarean Delivery Rate by Background Characteristics, Among All Mothers From All Survey Datasets (2002–2012)

	**OR (95% CI)**	**aOR (95% CI)**
Age, y		
15–19	1.00	1.00
20–24	0.85 (0.63–1.14)	0.82 (0.60–1.12)
25–29	1.01 (0.80–1.42)	0.97 (0.72–1.31)
≥ 30	1.47 (1.11–1.94)^**^	1.05 (0.78–1.41)
Place of Residence		
Urban	1.00	1.00
Rural	0.90 (0.82–0.99)*	0.96 (0.86–1.07)
Geographic Region		
Central	1.00	1.00
North	0.87 (0.80–0.94)^**^	0.91 (0.83–0.99)*
South	1.02 (0. 90–1.16)	1.03 (0.99–1.18)
Mother's Education		
No education	1.00	1.00
Primary	1.41 (1.08–1.85)*	1.33 (1.00–1.78)*
Secondary	1.01 (0.80–1.29)	0.96 (0.73–1.25)
Higher	1.11 (0.87–1.42)	0.79 (0.60–1.06)
Husband's Education		
No education	1.00	1.00
Primary	1.14 (0.82–1.59)	1.21 (0.84–1.73)
Secondary	1.02 (0.74–1.39)	1.16 (0.81–1.65)
Higher	1.22 (0.89–1.68)	1.17 (0.81–1.68)
Employment		
Unemployed	1.00	1.00
Employed	1.58 (1.43–1.75)^***^	1.34 (1.19–1.51)^***^
Place of Delivery		
Public sector	1.00	1.00
Private sector	1.17 (1.08–1.26)^***^	1.04 (0.95–1.14)
Wealth Index		
Poorest	1.00	1.00
Poorer	0.94 (0.84–1.05)	0.94 (0.84–1.06)
Middle	1.20 (1.07–1.34)^***^	1.27 (1.13–1.43)^***^
Richer	1.30 (1.16–1.45)^***^	1.32 (1.16–1.51)^***^
Richest	1.47 (1.30–1.65)^***^	1.39 (1.20–1.62)^***^
Parity in Last 5 years		
1	1.00	1.00
2	0.86 (0.79–0.93)^***^	0.81 (0.75–0.88)^***^
≥ 3	1.04 (0.90–1.18)	0.82 (0.70–0.95)*
Age at First Birth, y		
12–18	1.00	1.00
19–24	1.10 (0.98–1.22)	1.14 (1.02–1.28)*
25–30	1.72 (1.52–1.94)^***^	1.75 (1.52–2.01)^***^
> 30	4.69 (3.93–5.61)^***^	4.58 (3.77–5.55)^***^
Birth Weight, g		
Normal (2500–3999)	1.00	1.00
Low/High (< 2500 or ≥ 4000)	1.55 (1.43–1.68)^***^	1.53 (1.40–1.66)^***^
Birth Multiplicity		
Singleton birth	1.00	1.00
Multiple birth	5.47 (4.38–6.82)^***^	5.60 (4.20–7.09)^***^

Abbreviations: aOR, adjusted odds ratio (for all covariates under analysis); CI, confidence interval; OR, odds ratio.

^*^P< .05; ^**^ *P<* .01; ^***^ *P*< .001.

## DISCUSSION

This study shows that in Jordan the cesarean delivery rate has increased steadily over the past decade by two-thirds, from 18% in 2002 to 30% in 2012, and was double the maximum population-based rate of 15% recommended by WHO.^10^ In Jordan, 99% of deliveries occur in hospitals^14^; thus, the cesarean delivery rates reported in the JPHFS (on which this study is based) reflect population-level rates since virtually all deliveries occur in facilities.

The current cesarean delivery rate of 30% in Jordan is double the threshold that WHO considers reasonable.

A similar study analyzing nationally representative population survey data from Jordan between 1990 and 2002 reported that the cesarean delivery rate was 8.5% in 1990 and 12.9% in 1997.^12^ In the current study, the 2012 cesarean delivery rate of 30.3% is 3.6 times higher than the 1990 rate of 8.5%.^12^ This increase is in line with consistent global increases in the cesarean delivery rate.^21^ Some of the increase may be justified, for example, due to better access to maternal health services^12^ and delayed age at marriage resulting in advanced age at birth, which may be associated with adverse pregnancy outcomes.^22^^,^^23^ However, the sizeable increases suggest that many cesarean deliveries might occur without any, or on questionable, medical indications.

Globally there has been an increase in demand for elective cesarean delivery. An estimated 4% to 18% of all cesarean deliveries worldwide are requested by mothers.^24^ In this study, from 2002 to 2012, the cesarean delivery rate in births that were likely low risk increased significantly over time after controlling for potentially confounding factors—among normal birth weight infants by 93% and among singletons by 92%.

After combining all 3 datasets and adjusting for all variables under analysis, the findings revealed that, on average, mothers who were employed had higher odds of cesarean delivery than unemployed mothers, and the cesarean delivery rate increased linearly with increasing wealth—both indicators of better economic status. This is consistent with findings from a study in Nepal.^20^ Mothers with higher economic status have a better chance of affording the expense of such a surgical procedure.

Cesarean deliveries increased among both public and private hospitals in Jordan but more substantially in public hospitals, and particularly at UTHs. The high rate of cesareans in UTHs was associated with higher cesarean delivery rates among apparently low-risk groups (singletons, normal birth weight infants) than among high-risk groups (multiples, low/high birth weight infants). Researchers have expected cesarean deliveries to increase particularly among the private sector in Jordan over time and have speculated that the gap between public- and private-sector cesarean delivery rates may be growing.^12^ However, in this study, the gap narrowed from a 4.0 percentage point difference (*P* = .004) in 2002 to 2.8 percentage points (*P* = .02) in 2012 (Table 2). The overall trend in cesarean deliveries between public and private hospitals from 2002 to 2012 was not significant (*P* = .53). This new finding differs from what was previously reported in Jordan.^12^

The gap in the cesarean delivery rate between public and private hospitals in Jordan is narrowing due to rising rates in public hospitals, particularly in university teaching hospitals.

In Jordan, there are 2 UTHs: the UTH of the University of Jordan and the King Abdullah University Hospital (KAUH) of the Jordan University of Science and Technology. A number of factors could potentially explain the higher rate of cesarean deliveries in UTHs in Jordan. First, the KAUH, which is the largest UTH, opened to the public in November 2002 and dramatically increased access to advanced medical procedures, including cesarean deliveries, for a large segment of the Jordanian population, specifically for those living in rural areas. In addition, the government initiated a health reform agenda in 2005, which included improving health insurance coverage, that could have increased access to government hospitals.^25^ Finally, gynecology and obstetrics residents at UTHs may be more inclined to perform cesarean deliveries for the sake of practicing the surgical procedure, potentially increasing the rate of elective cesareans.

This study used data from population-based surveys that are usually conducted every 5 years. Collecting and monitoring such data in routine health information systems (RHIS) on a more regular basis (for example, annually or semiannually) could help policy makers and health professionals develop and enforce strategies to control the increase in the cesarean delivery rate, particularly cesarean deliveries without any medical indication.

A number of clinical, psychosocial, and structural strategies have been used to reduce the likelihood of cesarean deliveries, even among those who may have a medical indication.^26^ For example, a systematic review demonstrated that external cephalic version (a procedure used to turn the fetus from a breech or transverse position into a vertex, or head-down, position before labor begins) and vaginal birth after a previous cesarean were effective at reducing cesarean delivery rates.^26^ In addition, one-on-one trained support during labor has been shown to reduce cesarean delivery rates.^26^

A number of structural strategies have been shown to be effective in reducing cesarean deliveries.

Initiatives to raise awareness among the public and among health professionals about the adverse maternal outcomes associated with cesarean deliveries and advantages of vaginal delivery are also urgently needed to halt the steady increase in the cesarean delivery rate. Health professionals should give mothers full information about risks associated with cesarean delivery. Other interventions, such as midwifery training and education and establishment of birthing centers, could also help encourage mothers to deliver vaginally.

### Strengths and Limitations

This study has 3 major strengths. First, analysis was based on data collected from nationally representative samples by accredited and reliable official entities using comparable sampling procedure and inclusion criteria. Second, the probability of recall bias is low since women who had a cesarean delivery would not easily forget the mode of delivery given its surgical nature, particularly for the last birth. A recent study showed that DHS data on cesarean deliveries are sufficiently reliable for national and global monitoring purposes.^27^ Third, stratification according to the type of hospital provided more insights about the substantial contribution of UTHs to the rising cesarean delivery rate in Jordan.

Limitations include that samples were collected through a cross-sectional design that limits the causality pathway with regard to the drivers of increased cesarean delivery rates and that the data did not include information about whether the cesarean deliveries were performed under medical indications or based solely on maternal demand. Instead, this study used only surrogate markers of what could potentially have been births at low or high risk of medically necessary cesarean delivery.

## CONCLUSION

The cesarean delivery rate increased in Jordan from 2002 to 2012; the last survey in 2012 showed that the rate was double the maximum threshold recommended by WHO. The cesarean rate was higher among apparently low-risk mothers, and was particularly higher at UTHs than in private or government hospitals, which suggests that many cesarean deliveries may have been performed without medical indications. Delayed maternal age at first birth, low/high birth weights, multiple births, employment of mothers, and greater wealth were the main predictors of cesarean delivery in Jordan. More vigilant monitoring is needed to reduce unnecessary cesarean deliveries in what appear to be low-risk mothers, and in those delivering at UTHs in particular.
